# The role of gut microbiota in the onset and development of sepsis and its therapeutic potential: mechanisms and research progress

**DOI:** 10.3389/fmicb.2025.1718549

**Published:** 2025-12-15

**Authors:** Zhiyang Tian, Changhui Li

**Affiliations:** Department of Critical Care Rehabilitation, The Affiliated Hospital of Liaoning University of Traditional Chinese Medicine, Shenyang, China

**Keywords:** sepsis, gut microbiota, immune system, inflammatory dysregulation, organ failure

## Abstract

Sepsis is a life-threatening disease triggered by infection-induced immune dysregulation, characterized by multi-organ dysfunction, and is one of the leading causes of death among critically ill patients worldwide. Recent studies have shown that gut microbiota (GM) imbalance plays a crucial role in the progression of sepsis. This review identifies the core mechanisms of GM imbalance: it disrupts the integrity of the intestinal mucosal barrier, induces bacterial and endotoxin translocation, activates systemic inflammatory responses, and forms a vicious cycle of “gut-organ” cross-damage, becoming a key driver of sepsis-associated multi-organ dysfunction. Existing research has confirmed that microbiota modulation strategies, such as probiotic supplementation and fecal microbiota transplantation (FMT), have potential therapeutic value. However, due to issues like strain specificity, lack of standardized protocols, and insufficient clinical evidence, the clinical translation of these strategies still faces significant barriers. Therefore, future research should focus on the identification of sepsis-specific GM core functional biomarkers, the development of personalized combined regulatory strategies, and the advancement of targeted delivery technologies. Multi-center large-scale clinical trials are needed to validate their efficacy and safety, providing innovative solutions for precision treatment of sepsis.

## 1 Introduction

Sepsis is a life-threatening organ dysfunction caused by a dysregulated immune response to infection. It is often accompanied by Systemic Inflammatory Response Syndrome (SIRS) and is manifested by the failure of multiple organ systems (Angus and van der Poll, 2013). The occurrence of sepsis is closely related to the type of infection source, the host’s immune status (van der Poll et al., 2021), and its response to the infection, making it one of the leading causes of death worldwide. According to a report from the World Health Organization (WHO), the incidence and mortality rates of sepsis remain high (La Via et al., 2024). The pathogenesis of sepsis is complex, involving immune activation, dysregulated inflammatory response, microcirculatory disturbances, and organ failure (Rossaint and Zarbock, 2015). Although there have been advancements in early diagnosis and treatment, clinical management still faces significant challenges. This is mainly due to the lack of effective treatment strategies and early screening methods. Antibiotic resistance and insufficient personalized treatment further exacerbate the difficulty of managing sepsis.

The GM is a diverse community of microorganisms, including bacteria, fungi, and viruses, that reside in the human gut and play several important roles (Hou et al., 2022). They help break down food, promote nutrient absorption, and maintain immune system balance by interacting with immune cells to prevent immune dysregulation. Additionally, the GM is crucial for maintaining the intestinal barrier, protecting the gut from pathogen invasion (Thursby and Juge, 2017). It also participates in vitamin synthesis and toxin breakdown, having a profound impact on overall health. Recent studies have shown that the occurrence and progression of sepsis are closely linked to dysbiosis of the GM. Sepsis patients often exhibit significant changes in their GM, characterized by a reduction in microbial diversity or an overgrowth of harmful microorganisms. This imbalance not only affects the normal function of the immune system, leading to immune response disorders, but also disrupts the gut barrier, increasing intestinal permeability. When intestinal permeability is elevated, endogenous gut bacteria, bacterial products, and toxins are more easily able to enter the bloodstream. This triggers a systemic inflammatory response, which further exacerbates the development and progression of sepsis (Luan et al., 2024). Furthermore, dysbiosis of the GM may also exacerbate the inflammatory response by altering the immune environment and metabolic processes within the gut, worsening the symptoms of sepsis (Kullberg et al., 2021). Therefore, investigating the mechanisms by which the GM influences sepsis is crucial for a deeper understanding of the pathological process of sepsis. This research is essential for identifying new therapeutic targets and developing more effective treatment strategies. Modulating the GM or repairing the gut barrier function may offer new directions for the treatment of sepsis.

## 2 The role of GM in the occurrence and development of sepsis

### 2.1 Composition and function of the GM

The GM of healthy individuals is composed of a variety of microorganisms, including bacteria, archaea, fungi, and viruses (Rinninella et al., 2019). Among the bacteria, the phyla *Bacteroidetes* and *Firmicutes* dominate the microbial composition (Fujisaka et al., 2023). In addition, *Lactobacillus, Bifidobacteria*, and other microorganisms play essential roles in maintaining host health. Other microbial communities, such as archaea (e.g., *methanogens*), further promote bacterial metabolism and inhibit the generation and accumulation of harmful substances by consuming hydrogen gas in the gut (Moissl-Eichinger et al., 2018). Fungi (e.g., *Candida*) play a crucial role in maintaining the stability and balance of the GM, as well as reducing the risk of infections (Liu et al., 2025). Viruses in the body, primarily bacteriophages, regulate the structure and function of bacterial communities through interactions with gut bacteria (Sanz-Gaitero et al., 2019). The GM, primarily composed of bacteria, maintains stability and modulates host physiological functions through the synergistic interactions between different microbial groups. The GM performs multiple critical functions in sustaining host health. Firstly, GM plays a crucial role in digestion by assisting in the breakdown of indigestible components in food and synthesizing vitamins and short-chain fatty acids (SCFAs). It also regulates energy balance and promotes gut health (Rowland et al., 2018). Additionally, the GM interacts with the immune system to help maintain immune tolerance, prevent excessive immune responses against the body’s own tissues, and enhance the body’s defense against external pathogens. Moreover, GM regulates gut barrier function, preventing harmful substances from entering the body, suppressing intestinal inflammation, and ensuring the structural and functional integrity of the gut (Di Vincenzo et al., 2024). The relationship between the GM and the host is mutually beneficial, working together to maintain the health and balance of the body.

The balance and diversity of the GM are crucial for the overall health of the host during the occurrence of severe infections such as sepsis. A clinical cohort study conducted by relevant scholars analyzed the GM of 14 sepsis patients. The results showed that upon ICU admission, the dominant microbiota consisted of *Bacteroidetes*, *Firmicutes*, and *Proteobacteria*. After treatment, the GM diversity significantly decreased. *Veillonella* and *Ruminococcus* were identified as signature bacteria for non-severe and severe patients, respectively, while *Enterococcus* was the signature bacterium at discharge. Additionally, liver function was found to be associated with the abundance of GM, further indicating a significant reduction in GM diversity in sepsis patients (Liu et al., 2023). A study found that the GM of critically ill septic patients significantly differs from that of non-septic patients. There is a notable increase in the abundance of inflammation-associated microorganisms, such as *Prevotella*, *Clostridium*, and *Bifidobacterium* (Piccioni et al., 2024). A multi-center study found that the GM of critically ill patients, including those with sepsis, undergoes significant changes during ICU stays. In septic patients, the abundance of inflammation-related bacteria, such as *Parabacteroides*, *Fusobacterium*, and *Bifidobacterium*, increases, and women and older patients are at higher risk for sepsis. Additionally, microbial diversity decreases during ICU stays, and pathogenic bacteria (such as *Enterococcus* species) increase in the gut of deceased patients (Agudelo-Ochoa et al., 2020). The findings of these studies indicate that the GM not only plays a crucial role in the regulation of sepsis but may also serve as a potential biomarker for its early prediction. Therefore, monitoring and modulating the GM could provide new approaches and strategies for the early diagnosis and treatment of sepsis.

### 2.2 The impact of GM dysbiosis on the neuro-immune system in sepsis patients

Gut microbiota dysbiosis refers to a deviation of the gut microbial community structure and metabolic pathways from the normal physiological state. It is typically manifested as a reduction in beneficial bacteria, such as *Lactobacillus* and *Bifidobacterium*, an increase in pathogenic bacteria, such as *Escherichia coli* and *Aspergillus*, or a decline in microbial diversity (Shen et al., 2025).

#### 2.2.1 Immunoregulation

The gut is the largest immune organ in the body, with about 70% of immune cells distributed within it. The associated lymphoid tissues of the gut are the core components of its immune system, rich in various immune cells such as lymphocytes and macrophages (Chassaing et al., 2014). The GM interacts with the host immune system to maintain immune tolerance, preventing excessive activation of the immune system that could attack self-tissues. At the same time, the GM regulates immune responses and promotes the maintenance of immune tolerance through interactions with intestinal epithelial cells, immune cells (such as macrophages, dendritic cells, etc.), and endothelial cells (Yoo et al., 2020). The immune response in sepsis is typically divided into an early immune activation phase and a later immune suppression phase (Delano and Ward, 2016). During the early phase, the immune system is excessively activated, with the large release of inflammatory factors, triggering the immune regulatory mechanisms of sepsis and further intensifying the inflammatory response. The body actively mobilizes immune cells to enhance the immune response (Jarczak and Nierhaus, 2022). However, when the immune system fails to adequately address the inflammation caused by severe infection and does not promptly clear the pathogens, immune imbalance occurs. This leads to the impairment of immune cell function and survival, ultimately resulting in the immune suppression phase (Bender et al., 2025). In sepsis, the immune regulatory role of the GM is both complex and crucial. Timothy Buchman’s research team discovered through experiments that CD4^+^ T lymphocytes are key factors in regulating intestinal epithelial cell survival and host prognosis in sepsis. This finding challenges the limitations of traditional immunosuppressive theories of sepsis and provides an innovative theoretical foundation for targeting CD4^+^ T cells or the Bcl-2 pathway in the treatment of sepsis (Stromberg et al., 2009). The GM regulates the host’s immune response through various pathways. In the immune suppression state, the impaired immune function of septic patients may lead to the excessive proliferation of pathogens. This allows normally harmless opportunistic pathogens and resident pathogens to disrupt the host’s immune defenses and alter the balance of the GM (Gao et al., 2025). Research has shown that an imbalance in the GM leads to a disruption in the ratio of beneficial bacteria to pathogenic bacteria, which in turn activates relevant signaling pathways in the body. These pathways promote the release of pro-inflammatory cytokines such as interleukin-1 beta (IL-1β), interleukin-6 (IL-6), and tumor necrosis factor-alpha (TNF-*α*), ultimately exacerbating intestinal inflammation and damaging the intestinal barrier function (Ma et al., 2022). Further studies have indicated that various metabolites, bacteriocins, and certain bacteria produced by the GM can activate the immune system by binding to epithelial cells through the expression of specific antigens. This process induces metabolic changes in the gut and other organs, playing a significant role in the development of sepsis (Liu et al., 2022). Microglia are immune cells in the central nervous system, typically responsible for maintaining neural health and defending against foreign invasions. However, immune dysregulation caused by sepsis can lead to their overactivation, resulting in an inflammatory response (Borst et al., 2021). Studies have shown that the GM influences microglia through metabolites and bacteriocins, inducing the production of specific antigens that trigger T lymphocyte responses. This not only activates microglia but also promotes their proliferation, leading to the accumulation of cells in neural tissue. This exacerbates the inflammatory response, potentially causing neuronal damage and increasing the mortality rate in sepsis patients (Bettag et al., 2023). Through this mechanism, the GM not only affects the nervous system but may also worsen damage to other organs, creating a widespread immune dysregulation network (Moraes et al., 2021).

Immune dysregulation induced by GM imbalance often manifests in preterm infants and young children. Graspeuntner and colleagues conducted a prospective single-center case–control study, including 51 infants with late-onset sepsis (LOS) under 90 days of age and 40 healthy controls (Graspeuntner et al., 2025). The study employed conventional microbiological diagnostics, flow cytometry to analyze peripheral blood lymphocyte subsets, and 16S rRNA sequencing to analyze the GM. The results indicated that infants with LOS under 90 days exhibit a characteristic disruption in GM-immune interactions. The reduction of *Bifidobacterium longum* and the associated loss of immune regulation may be potential risk markers for LOS. Similar studies have shown that the development of GM in preterm infants is disrupted by factors such as cesarean section, high-risk NICU environments, antibiotic misuse, and interruption of breastfeeding. These factors result in low microbial diversity, reduced probiotics, and an increase in pathogenic bacteria. This imbalance promotes systemic inflammation, increases the severity of sepsis, and may lead to organ dysfunction (Erickson et al., 2025). In addition, a study by Luo et al. showed that sepsis patients exhibit innate immune deficiencies, such as a reduced proportion of NK cells and plasmacytoid dendritic cells (pDCs). The study also highlighted adaptive immune suppression, including T cell exhaustion. The diversity of the GM is significantly reduced, and the enrichment of *B. salyersiae* is closely associated with impaired NK cell function (Luo et al., 2025). These studies suggest a close link between immune dysregulation in sepsis and changes in the GM. By integrating immune biomarkers and microbiota characteristics, the diagnostic accuracy and prognosis assessment of sepsis can be improved.

#### 2.2.2 Neural regulation

In recent years, the impact of the GM on central nervous system diseases through the microbiome-gut-brain axis has gradually become a research hotspot (Gershon and Margolis, 2021). The gut is referred to as the “second brain,” and its microbiome connects to the central nervous system via the vagus nerve, influencing various brain functions (Ochoa-Repáraz and Kasper, 2016). In sepsis patients, the brain is typically the first organ to be affected, and sepsis-associated encephalopathy (SAE) has become one of the severe complications secondary to sepsis (Ren et al., 2020). In the CLP-induced SAE mouse model, studies have shown a significant reduction in bacteria related to host cognitive function and the synthesis of SCFAs, such as *Bacteroides*, *Collinsella*, *Firmicutes*, and *Prevotella*. The decrease in these bacteria leads to a reduction in SCFA levels, which affects the gut-brain axis, triggers neuroinflammation, and exacerbates cognitive impairment (Li et al., 2023). As a key regulator of the gut-brain axis, SCFAs alleviate sepsis-associated brain damage by inhibiting excessive activation of microglia and reducing the release of pro-inflammatory cytokines. A study found that in SAE mice, the levels of SCFAs significantly decreased, and the related beneficial bacteria were reduced. Supplementation with SCFAs increased SCFA levels, alleviated cognitive dysfunction, and reduced neuroinflammation. The levels of inflammatory factors IL-1β, IL-6, and TNF-*α* were lowered, and SCFAs exerted protective effects through the GPR43 receptor, improving cognitive function and anti-inflammatory effects (Liao et al., 2022). Early studies have shown that in sepsis animal models and patients, gut microbes, such as *Enterobacteriaceae* and *Bacteroides*, can translocate through the blood–brain barrier to the brain. This suggests that microbial translocation may be a key factor in driving brain infection responses (Lynch et al., 2023). Further research has revealed that gut dysbiosis and microbial translocation exacerbate neuroinflammation, becoming a major driver of SAE. Once gut microbes enter the brain via the bloodstream, they can activate immune responses, leading to neuronal damage and cognitive impairment (Chidambaram et al., 2022). Moreover, the metabolic products of gut microbes may intensify the inflammatory response, further impairing brain function. These findings highlight the critical role of GM dysbiosis in the development of SAE, offering new insights for early prevention and treatment strategies.

### 2.3 The disruption of the gut barrier system and its association with sepsis

The intestinal barrier system is composed of biological, mechanical, chemical, and immune defenses that work in synergy to maintain the stability of the intestinal environment and prevent pathogen invasion (Vancamelbeke and Vermeire, 2017). During the pathological process of sepsis, the intestinal barrier plays a dual role. Firstly, as the primary target organ affected after infection, the integrity of the intestinal barrier may be compromised by factors such as GM imbalance. For instance, lipopolysaccharides can downregulate the expression of tight junction proteins (TJs), increase intestinal permeability, and subsequently lead to intestinal injury. Secondly, damage to the intestinal barrier can serve as a key factor in the development of multiple organ dysfunction syndrome (MODS) (Assimakopoulos et al., 2018). Once the intestinal barrier is compromised, bacteria can spread to other organs via the bloodstream and lymphatic system. This triggers a systemic inflammatory response through the “gut-axis” pathway, further promoting the progression of organ failure (Nagpal and Yadav, 2017). The GM and intestinal mucosa together form the biological barrier. The mucosal microbiota primarily consists of strict anaerobes such as *Bifidobacterium* and *Lactobacillus*, which play a key role in maintaining intestinal physiological functions (Okumura and Takeda, 2024). Studies have shown that dysbiosis and the accumulation of its metabolic products may exist even before the onset of sepsis. In critically ill sepsis patients, the GM undergoes rapid alterations, characterized by a significant reduction in microbial diversity and a decrease in resident symbiotic bacteria. This is accompanied by an overgrowth of potential pathogens such as *Enterococcus* and *Staphylococcus*, leading to the onset of an inflammatory storm (Origüela and Lopez-Zaplana, 2025). The mechanical barrier of the intestinal barrier is located in the extracellular spaces formed by intestinal mucosal epithelial cells and tight junctions, creating a fundamental physical barrier that can block potential pathogens (Suzuki, 2013). TJs are a crucial component of the intestinal physical barrier, regulating the diffusion of substances. They also effectively prevent the penetration of antigens, bacterial toxins, and pathogens, thereby promoting the onset of secondary organ dysfunction in sepsis patients. Furthermore, the integrity of tight junctions is vital for the repair process of intestinal epithelial cells (Chelakkot et al., 2018; Arumugam et al., 2025). The normal function of the intestinal mucosa relies on the involvement of chemical cofactors, which maintain the integrity of the chemical barrier by promoting epithelial cell turnover (Gieryńska et al., 2022). Diamine oxidase (DAO) is a highly active intracellular enzyme primarily found in the upper villi of the intestinal mucosa, and its activity is closely associated with intestinal mucosal injury. A decrease in DAO activity usually indicates the worsening of mucosal damage, and DAO activity is positively correlated with intestinal mucosal permeability. Therefore, DAO activity can serve as an important biomarker for assessing the integrity and degree of damage to the intestinal mucosa (Thompson et al., 1992). Modern research suggests that secretory immunoglobulin A (sIgA) is a key component of the intestinal mucosal immune barrier, providing multiple defensive functions. When sIgA secretion is reduced, its ability to encapsulate bacteria weakens, leading to a diminished defense against toxins in the intestine and increasing the risk of enteric infections (Pietrzak et al., 2020). Additionally, reduced sIgA levels may indirectly promote the generation of inflammatory factors, exacerbating intestinal inflammation and further damaging the intestinal mucosa (Wang et al., 2020). In summary, once the intestinal barrier is compromised, pathogenic bacteria and endotoxins can invade the body and enter the bloodstream, serving not only as a critical trigger for sepsis but also potentially accelerating the process of organ dysfunction.

### 2.4 The regulation of systemic inflammatory response induced by sepsis by the GM

**Figure 1:**
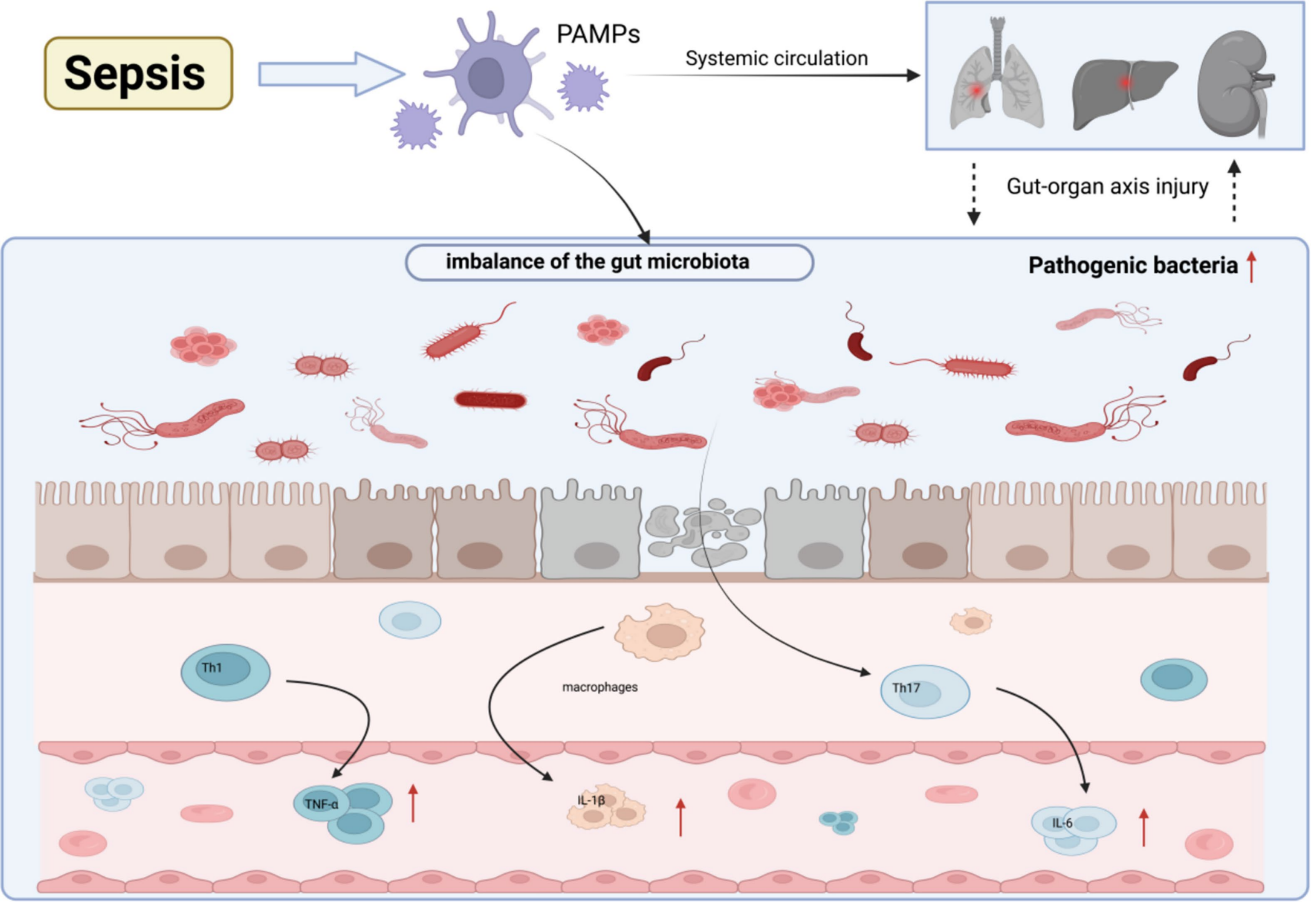
Sepsis induces GM dysbiosis, and the excessive release of inflammatory factors exacerbates multiple organ failure.

Sepsis is a severe infectious disease characterized by a cascade of inflammatory factors. When the body suffers from infection or injury, an inflammatory response is triggered as a natural mechanism, with the primary function of protecting the body and promoting tissue repair (Zhang and Ning, 2021). If the inflammatory response is excessively activated or loses regulation, it can lead to tissue damage, organ dysfunction, and ultimately multi-organ failure (Figure 1). Recent studies have shown that there is a complex interaction between the GM and systemic inflammatory response. The GM modulates the host’s immune system, influencing the onset and progression of systemic inflammation (Yang and Cong, 2021). In septic shock, pathogen-associated molecular patterns (PAMPs) and damage-associated molecular patterns (DAMPs) activate the immune system, triggering a SIRS. During this process, pro-inflammatory cytokines such as TNF-*α* and IL-1β are released in large amounts, leading to damage of endothelial cells and intestinal epithelial cells (Moriyama and Nishida, 2021). This disrupts the intestinal barrier, increases intestinal permeability, and facilitates the entry of bacteria and endotoxins into the bloodstream and lymphatic system. Chemokines such as interleukin-8 (IL-8) play an important role in this process by recruiting neutrophils to the site of inflammation, exacerbating the intestinal inflammatory response (Russo et al., 2014). As a result, intestinal permeability further increases, and pathogens and endotoxins enter the bloodstream through the gut, thereby amplifying the systemic inflammatory response.

When endotoxins enter the liver via the portal vein, they activate Kupffer cells (liver macrophages), which release more IL-1β and IL-6, further intensifying the inflammatory response (Kessoku et al., 2021). Simultaneously, the liver’s detoxification capacity weakens, leading to persistent endotoxemia and trapping the body in a vicious cycle of “gut-origin sepsis.” Additionally, the inflammatory response in sepsis leads to intestinal hypoxia and pH changes. These alterations in the internal environment stimulate the overgrowth of conditionally pathogenic bacteria, such as *Enterobacteriaceae*. Meanwhile, beneficial GM, such as *Bifidobacterium* and *Lactobacillus*, decrease, resulting in an imbalance of the GM (Fay et al., 2017). This imbalance exacerbates the damage to the intestinal barrier, increasing the risk of bacterial and harmful substances entering the bloodstream. As the intestinal barrier function further deteriorates, the clinical manifestations of sepsis become more severe, and the condition becomes increasingly complex.

In septic shock, PAMPs activate the innate immune system, disrupting the GM homeostasis and triggering dysbiosis. The excessive release of pro-inflammatory factors such as TNF-*α* and IL-1β damages vascular endothelial and intestinal epithelial cells, impairing the intestinal barrier. This increases intestinal permeability and promotes the translocation of bacteria and endotoxins. This may ultimately lead to MODS and death in septic shock patients.

### 2.5 GM imbalance drives sepsis-associated organ failure

**Figure 2:**
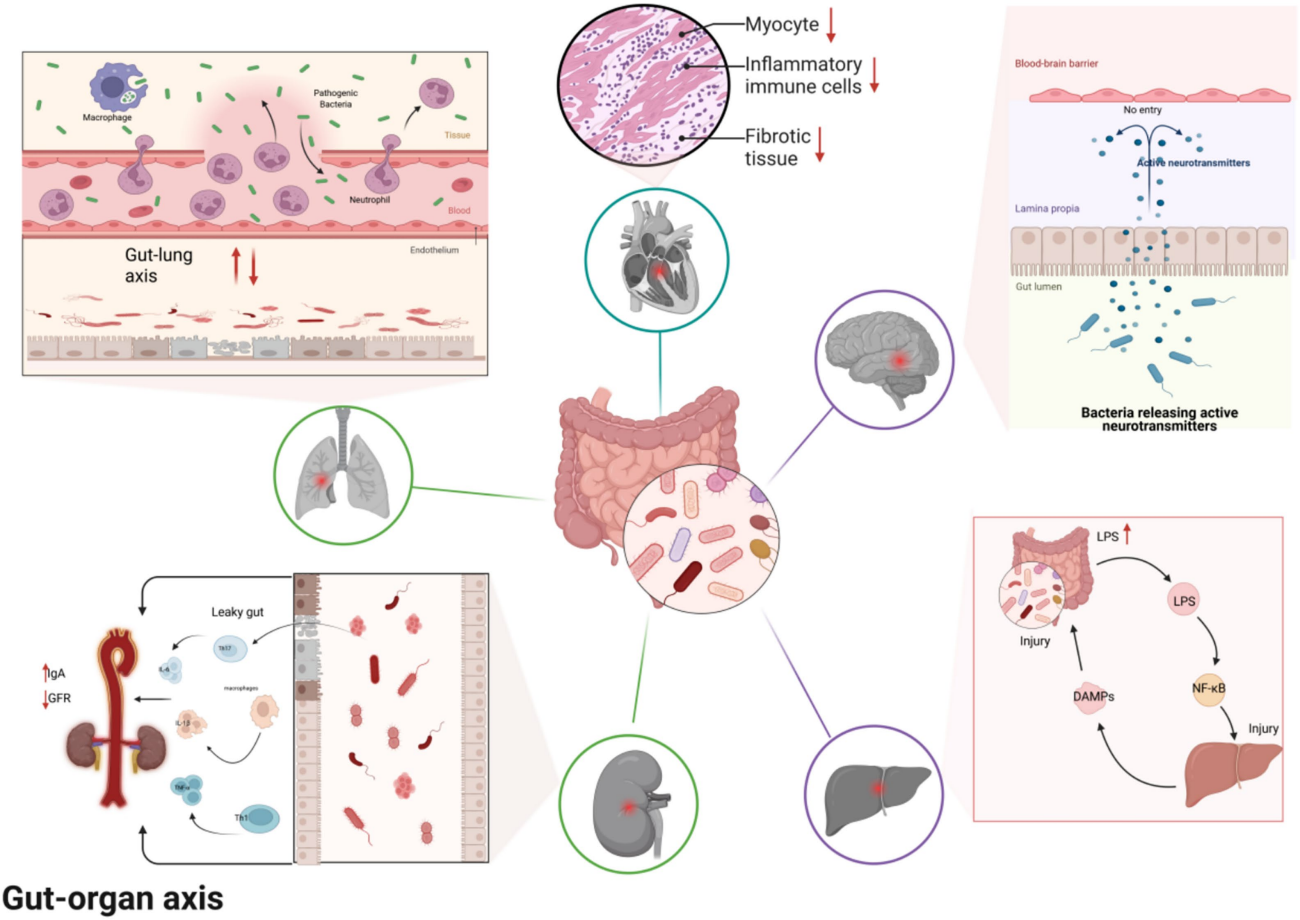
GM imbalance drives sepsis-related organ failure.

In the pathological process of sepsis, the GM imbalance-induced dysfunction of the gut-axis is a key link connecting the gut with multi-organ damage (Borges and Bento, 2024). As the largest immune and metabolic organ in the human body, the gut plays a vital role in maintaining health. It establishes close pathophysiological connections with organs such as the lungs, liver, kidneys, brain, and heart through the complex network of the “gut-organ axis” (Figure 2). The core mechanism of sepsis-associated organ injury can be attributed to the multi-organ damage process triggered by GM dysbiosis. First, GM imbalance disrupts the integrity of the intestinal mucosal barrier, leading to the translocation of endotoxins (such as lipopolysaccharides) and bacteria, which lays the foundation for the spread of pathological signals (Piccioni et al., 2024). Next, the translocated bacteria and endotoxins enter the circulatory system, activating immune cells and releasing a large number of pro-inflammatory cytokines, resulting in a systemic inflammatory response and cross-activation of immunity, further exacerbating the systemic inflammatory burden (Sun et al., 2020). Finally, inflammatory signals and metabolic by-products spread to various organs through the blood, lymph, or neural pathways, directly damaging the function of target organs and aggravating intestinal barrier injury, thereby forming a vicious cycle of “gut-organ” mutual damage. This ultimately drives the progression of organ dysfunction and failure (Ziesmann and Marshall, 2018).

In sepsis, the gut interacts closely with key organs such as the lungs, liver, kidneys, brain, and heart through the “gut-organ axis,” with dysfunction exacerbating multi-organ damage. Gut dysbiosis disrupts the intestinal barrier, releasing endotoxins and pro-inflammatory factors that trigger inflammation and damage in the lungs, liver, kidneys, brain, and heart, forming a vicious cycle that ultimately leads to multi-organ failure.

#### 2.5.1 Gut-lung axis: inflammation dissemination and immune cross-activation

Both the lungs and the intestines originate from the endodermal foregut region. Their mucosal structures belong to the mucosal immune system, and through microbiota, metabolic products, and immune regulatory signals, they achieve bidirectional regulation, forming the “gut-lung axis” (Faure and de Santa, 2011). The lungs are not a sterile environment. In patients with sepsis or acute respiratory distress syndrome (ARDS), intestinal bacteria can be found in the lung microbiota, and this presence is associated with the severity of inflammation. Conversely, lung dysbiosis can also affect the intestinal microbiota, creating a vicious cycle of “lung-intestine” dysbiosis (Xiao and Xiao, 2019). Robert Dickson and colleagues, through metagenomic sequencing, have for the first time confirmed that the alveolar microbiota in patients with sepsis and ARDS is enriched with Bacteroides and other gut-derived microbial communities. This study provides a comprehensive understanding of the pulmonary migration mechanism via hematogenous or lymphatic pathways, offering a novel therapeutic strategy targeting the gut-lung axis microbiota for the treatment of sepsis-associated ARDS (Dickson et al., 2016). Some studies have focused on sepsis caused by Gram-negative bacilli, particularly analyzing the dynamic changes in the lung and GM. The research found that as sepsis progresses, changes in the GM are closely related to the severity of pneumonia. As pneumonia worsens, corresponding changes in the GM also occur (Duan et al., 2023). The gut-lung axis forms a bidirectional regulatory mechanism through the mucosal immune network, blood circulation, and the lymphatic system (Song et al., 2024). Endotoxins and pro-inflammatory factors released from intestinal barrier damage enter the lungs, activating alveolar macrophages and neutrophils, triggering pulmonary inflammation. At the same time, cytokines secreted by gut-associated lymphoid tissue (GALT) increase alveolar capillary permeability, which may ultimately lead to ARDS. Conversely, lung inflammation worsens gut barrier damage, creating a “gut-lung mutual damage” cycle, contributing to respiratory failure in sepsis (Mohammad and Thiemermann, 2020).

#### 2.5.2 Gut-liver axis: metabolic disorders and detoxification dysfunction

The liver receives blood from the intestines through the portal vein, thereby establishing a close anatomical and physiological connection with the intestines (Sibulesky, 2013). Under normal physiological conditions, about three-quarters of the intestinal blood flows to the liver via the portal vein. This blood carries beneficial metabolic products and regulatory mediators from the intestines, which help maintain the structure and function of the liver. However, during the pathological process of sepsis, the gut and liver form a vicious cycle through the gut-liver axis, known as the “mutual damage” mechanism. This manifests as a bidirectional effect: first, intestinal barrier dysfunction impairs the absorption and transport functions of intestinal epithelial cells, leading to deficiencies in nutrients such as vitamin C and zinc, which directly affect liver structure and function (Nicoletti et al., 2019; Shahid et al., 2024). Furthermore, liver injury, in turn, promotes the destruction of the intestinal barrier. Liver damage leads to immune and coagulation dysfunction, and the death of hepatocytes releases DAMPs, which impair the intestinal immune barrier (Hora and Wuestefeld, 2023). Additionally, liver injury reduces the synthesis of LPS-binding proteins, resulting in elevated LPS concentrations in the blood, which directly damage the intestinal barrier. Liver dysfunction also leads to insufficient synthesis of coagulation factors, triggering systemic microcirculatory disturbances and further exacerbating intestinal ischemic injury (Intagliata et al., 2018). The liver’s involvement in bile acid metabolism is closely linked to the conversion activities of the GM. Once the GM becomes imbalanced, it disrupts the homeostasis of the bile acid pool, increasing the metabolic burden on the liver. This may ultimately lead to liver failure and further exacerbate the functional disorder of the gut-liver axis (Shen et al., 2025).

#### 2.5.3 Gut-kidney axis: circulatory disturbances and toxin accumulation

The gut-kidney axis exerts its influence through various interactions, including hemodynamics, inflammatory mediators, and metabolic products. The systemic inflammatory response triggered by GM imbalance leads to vasoconstriction in the renal vessels, which in turn reduces renal blood flow and glomerular filtration rate (Di Vincenzo et al., 2024). Additionally, After intestinal barrier damage, endotoxins and bacterial translocation activate renal immune cells, inducing local inflammation and oxidative stress, thereby accelerating the onset of acute kidney injury (AKI) (Kunst et al., 2023). The accumulation of metabolic waste products (such as urea and creatinine) due to renal failure further damages the integrity of the gut barrier, increasing intestinal permeability, and forming a “gut-kidney toxicity cycle” (Rosner et al., 2021). This cycle is also one of the core factors accelerating the progression of renal dysfunction in sepsis patients.

#### 2.5.4 Gut-brain axis: neuroregulatory imbalance and the occurrence of related encephalopathies

The gut-brain axis achieves bidirectional communication through the autonomic nervous system, neuroendocrine signaling, and metabolic products of the GM (Carabotti et al., 2015). When the GM is dysregulated, excessive pro-inflammatory factors can cross the blood–brain barrier, activating microglial cells and triggering neuroinflammation (Parker et al., 2020). At the same time, neurotoxic substances such as kynurenine are produced from tryptophan metabolism disturbances. These substances can affect the synthesis of central nervous system neurotransmitters, leading to SAE, which manifests as consciousness disturbances, cognitive decline, and other symptoms (Dehhaghi et al., 2019; Schwarcz and Stone, 2017). In animal experiments simulating sepsis, fluctuations in inflammatory cytokines in the hippocampus are significantly associated with the occurrence of cognitive dysfunction (Zhou et al., 2024). The GM imbalance induced by sepsis reduces the synthesis of neurotransmitters such as serotonin and dopamine, which play key roles in brain function and cognition. The abnormalities in the synthesis of these neurotransmitters may indirectly affect cognitive and behavioral functions through immune and inflammatory responses (Piccioni et al., 2024; Haak and Wiersinga, 2017). SCFAs, such as acetic acid, propionic acid, and butyric acid, are metabolic products produced by gut microbes during the fermentation of dietary fibers. They can regulate immune responses and neuronal activity, thereby influencing the health of the central nervous system (He et al., 2020). SCFAs help maintain neurological function and cognitive abilities by reducing intestinal inflammation, modulating the immune system, and promoting neuroprotective mechanisms (Ikeda et al., 2022). In patients with cognitive impairment caused by diabetes, Parkinson’s disease, and SAE, changes in SCFA levels are closely related to disease progression and cognitive decline. Studies suggest that low levels of SCFAs may exacerbate the symptoms of these diseases and impair neurological function (Prado and Pacheco, 2024). Therefore, improving GM or increasing SCFA synthesis may become an effective strategy for treating neurodegenerative diseases, especially in the early stages, providing significant protection for the brain.

#### 2.5.5 Gut-heart axis: myocardial injury and circulatory failure

An imbalance in the GM leads to the release of endotoxins and pro-inflammatory factors, which directly damage myocardial cells and reduce myocardial contractility through the bloodstream (Al Bander et al., 2020). Meanwhile, the accumulation of trimethylamine-N-oxide (TMAO), a metabolite produced by the GM, promotes atherosclerotic lesions in the coronary arteries, further exacerbating myocardial ischemia (Caradonna et al., 2025). Sepsis-induced intestinal ischemia–reperfusion injury activates systemic inflammatory responses, causing vasodilation and decreased blood volume. This increases both the preload and afterload on the heart, ultimately leading to heart failure (Feinman et al., 2010; Nadatani et al., 2018). When cardiac function is impaired, blood perfusion to the gut decreases, causing insufficient perfusion, which further aggravates the imbalance in the GM. This creates a vicious cycle in which GM dysbiosis and heart failure mutually reinforce each other, making treatment more challenging (Zhao et al., 2025). Therefore, the imbalance of the GM not only directly affects myocardial health. It also damages cardiac function by altering microbial metabolites and intensifying systemic inflammatory responses.

## 3 GM regulation strategies in sepsis treatment

### 3.1 Probiotics in GM reconstruction

The role of probiotics in GM reconstruction is not solely dependent on “supplementing beneficial bacteria.” Instead, it involves multidimensional interventions that collaboratively restore microbiome structure, metabolic function, and immune responses. This approach achieves targeted repair of microbiome structure, enhances colonization resistance, and promotes the restoration of microbiome diversity (Gao et al., 2022; Thipe et al., 2022). Probiotics, as important regulators of the GM, maintain the homeostasis of the intestinal microbiota through competitive colonization and the secretion of antimicrobial metabolites. Additionally, they activate GALT and regulate immune cell activity. As a result, probiotics enhance the overall immune defense capacity of the body (Wang et al., 2021). In the pathogenesis of sepsis, patients experience an excessive systemic inflammatory response, leading to a hypermetabolic state. At the same time, malnutrition causes insufficient energy supply to the gut, and abnormal activity of intestinal metabolic enzymes triggers metabolic disturbances. These factors exacerbate damage to intestinal epithelial cells and degradation of TJs, further impairing the already fragile gut barrier function, creating a vicious cycle of “dysbiosis – barrier dysfunction – amplified inflammation” (Wasyluk and Zwolak, 2021; Lewis et al., 2016). Studies have shown that, in addition to conventional anti-infective treatment, the inclusion of probiotics can effectively optimize the GM environment, improving intestinal metabolic function and indirectly alleviating the nutritional imbalance in sepsis patients (Petrariu et al., 2023). Paul Wischmeyer and colleagues, using a cecal ligation and puncture (CLP)-induced septic mouse model, found that *Lactobacillus rhamnosus* and *Bifidobacterium longum* significantly reduced mortality and decreased bacteremia. Additionally, these probiotics improved colonocyte apoptosis and proliferation, and downregulated systemic and colonic inflammatory cytokine levels. Furthermore, their effects were mediated through the AKT pathway and by downregulating the TLR2/TLR4-MyD88 signaling pathway. These findings provide new experimental evidence supporting the use of probiotics in the treatment of pediatric sepsis (Khailova et al., 2013). In current clinical treatment and basic research, *Lactobacillus* and *Bifidobacterium* species are the most widely applied and thoroughly studied probiotic strains (Shah et al., 2024). Several high-quality clinical studies have clearly demonstrated the benefits of probiotic adjunct therapy for critically ill patients in intensive care units (ICU). This includes patients with conditions such as sepsis and MODS. Probiotic therapy can significantly reduce the incidence of diarrhea caused by gut dysfunction and lower the risk of hospital-acquired infections, including urinary tract infections and secondary bloodstream infections. Probiotics have been particularly effective in reducing the risk of ventilator-associated pneumonia (VAP) (Lou et al., 2024; Takeaki, 2025). Probiotics play a crucial role in improving intestinal mucosal barrier function and modulating immune responses in diseases associated with dysbiosis, particularly certain strains such as *Bifidobacterium* and *Lactobacillus*, which are considered to have significant effects (Zheng et al., 2023). However, due to the strong strain-specificity, there is considerable heterogeneity in their efficacy, with different patient responses varying. Additionally, the colonization efficiency of probiotics is influenced by the host’s baseline microbiota, and there is currently a lack of long-term safety data, making it difficult to comprehensively assess the potential risks of prolonged use. In clinical practice, there is no established standard for probiotic dosages, and the presence of placebo effects in various studies further complicates the interpretation of results. Overall, the interactions between probiotics and pharmaceuticals remain insufficiently explored, and there is no consensus on how to select appropriate patient populations. Therefore, while probiotics show clinical potential in regulating GM, their application faces numerous challenges, and further research and clinical trials are urgently needed to define the optimal strategies for their use.

### 3.2 Management of antibiotics and GM dysbiosis

In the clinical diagnosis and treatment of sepsis patients, the use of broad-spectrum antibiotics has a dual effect. On one hand, these antibiotics eliminate a large number of indigenous bacteria in the gut, which exposes mucosal binding sites and promotes the adhesion and colonization of pathogenic bacteria in the intestine (Varney et al., 2022). On the other hand, carbapenem antibiotics can rapidly clear bacteria, leading to the release of bacterial endotoxins into the bloodstream. This process further exacerbates the intestinal inflammatory response, reduces intestinal tissue perfusion, and ultimately causes abnormal intestinal motility. This highlights that the use of broad-spectrum antibiotics is a primary trigger for GM dysbiosis (Armstrong et al., 2021). Ferrer et al. (2017) analyzed the effects of 68 antibiotics on the microbiota of different parts of the human body, including the skin, mouth, gut, and respiratory tract. The results showed that the changes in microbiota induced by these antibiotics were primarily concentrated in the gut. Furthermore, there were significant differences in how various GM responded to the antibiotics. Specifically, *β*-lactam antibiotics had little effect on *lactobacilli*, while lincomycin antibiotics showed no significant effect on *streptococci*. Although fluoroquinolones did not affect *Prevotella* species, they had a notable impact on *Staphylococcus* and *Escherichia coli*, indicating that the rational selection of antibiotics is a key principle in reducing GM imbalance. The study further pointed out that the action spectrum of most antibiotics is usually limited to a few specific genera. For instance, nitrofurantoin and azole antibiotics mainly affect *Bifidobacterium* and *Faecalibacterium* species, while sulfonamides primarily impact *Bacteroides* and *Faecalibacterium* species. These findings suggest that different antibiotics target distinct microbiota components in the gut.

### 3.3 FMT

Fecal microbiota transplantation involves transferring the fecal microbiota from a healthy donor into a patient’s gut. Within 24 h, this procedure can restore the patient’s GM diversity to healthy levels (Thanush and Venkatesh, 2023). The core mechanism of FMT is the infusion of live, functional microorganisms from the healthy donor’s stool into the recipient’s gut, which helps rebuild the imbalanced GM (Wang et al., 2019). FMT not only repairs the structural damage caused by antibiotics and reverses microbiota dysbiosis, but it also alleviates abnormal local inflammation in the gut by suppressing the release of inflammatory factors such as IL-18 and IL-1β (Ma et al., 2022). Previous studies have confirmed that FMT, in conjunction with SCFAs, holds significant potential as an adjunctive therapy to mitigate antibiotic-related adverse outcomes (Zhao et al., 2024). This potential is particularly evident in its ability to effectively reduce the mortality rate in mice subjected to long-term antibiotic interventions.

From the analysis of disease-associated mechanisms, it is evident that the gut plays a critical role as an “amplifier” in the progression of sepsis. When the gut is damaged and its barrier function is compromised, the GM can breach the intestinal mucosal defense, invade distant organs such as the lungs, liver, and kidneys, and proliferate extensively. This ultimately leads to a vicious cycle of “intestinal damage – microbiota translocation – exacerbation of systemic infection” (Takiishi et al., 2017). In this pathological process, FMT can exert a dual regulatory effect. On one hand, by restoring the diversity of the GM, it suppresses the colonization of pathogenic bacteria. On the other hand, by alleviating the immune suppression induced by sepsis, it enhances the host’s ability to combat infections, ultimately reversing the pathological progression (Yadegar et al., 2024; Hou et al., 2025). Clinical case reports have confirmed the feasibility of FMT in the treatment of sepsis: Four patients with refractory sepsis complicated by diarrhea achieved clinical remission after undergoing FMT. The GM barrier function was restored, and significant improvements in clinical outcomes were observed. These findings provide direct evidence for the potential application of FMT in sepsis treatment (Li et al., 2014; Li et al., 2015; Wei et al., 2016).

Undoubtedly, FMT has shown remarkable efficacy in restoring intestinal microbiota homeostasis and treating recurrent *Clostridium difficile* infection (rCDI), and has demonstrated potential in modulating the microbiota in inflammatory bowel disease and metabolic syndrome. These findings suggest that FMT may emerge as a key therapeutic direction in future research. However, the clinical application of FMT in sepsis still faces numerous challenges that need to be addressed. First, the individual compatibility of donor microbiota varies significantly, and the persistence of colonization is poor, which limits the stability of the treatment effect. There are notable inter-individual differences in GM dysbiosis in sepsis patients (Lankelma et al., 2017), and the adaptability of donor microbiota in the recipient’s gut varies greatly, leading to inconsistent treatment responses. Secondly, the efficacy of FMT in non-rCDI indications, including sepsis, remains inconsistent, which restricts its widespread clinical application. Although preliminary case studies show potential value, large-scale clinical trials specifically targeting sepsis are still lacking (Haak et al., 2018). Moreover, the optimal timing, dosage, and administration route of FMT for sepsis patients have not yet been determined. Third, safety issues continue to be a concern: At present, there are no unified standards for donor screening, and the risk of transmitting potential pathogens, such as undetected symbiotic or opportunistic pathogens, has not been fully mitigated. Furthermore, the relationship between microbiota activity (e.g., production of metabolic byproducts) and treatment outcomes remains inadequately defined. Future research should focus on three key areas: developing standardized donor compatibility criteria based on the microbiota characteristics of sepsis patients, utilizing advanced screening technologies (e.g., metagenomic sequencing) and standardized sample processing protocols to rigorously control pathogen transmission risks; precisely matching donor microbiota with recipient needs (e.g., combining SCFAs or targeted probiotics) to enhance microbiota colonization persistence and therapeutic outcomes. These studies will systematically address compatibility, safety, and therapeutic stability issues of FMT in sepsis treatment and provide crucial support for the precision and standardization of microbiota-targeted therapies, ultimately advancing their transition from clinical exploration to standardized application.

### 3.4 The derived components produced by microbiota metabolism

Short-chain fatty acids, as key metabolic products of the GM, play a dual regulatory role in the gut defense system. Firstly, they strengthen the structural integrity and functional stability of the intestinal immune barrier, building a “physical defense line” (Sankarganesh et al., 2025). Secondly, they enhance the ability to clear pathogens, reinforcing the “immune clearance defense line.” These two mechanisms work synergistically to boost the gut’s overall defense effectiveness (Wang et al., 2024). However, current research on SCFAs is limited, as the majority of studies rely on mouse models, and the specific therapeutic mechanisms of SCFAs in sepsis patients remain unclear. This unresolved area may become a key focus of future research. In addition to this, the absorption and utilization of flavonoids in the human body is metabolism-dependent—they need to be absorbed in the form of metabolic products (Chen et al., 2022). These compounds also regulate the metabolic activity of the GM, promoting the production of SCFAs, with the GM being a critical participant in this metabolic pathway. The exogenous supplementation of SCFAs and flavonoids has shown potential therapeutic value in sepsis treatment, possibly offering a new treatment approach. However, this potential is still distant from actual clinical application and requires further clarification through large-scale, randomized controlled clinical trials.

**Figure 3:**
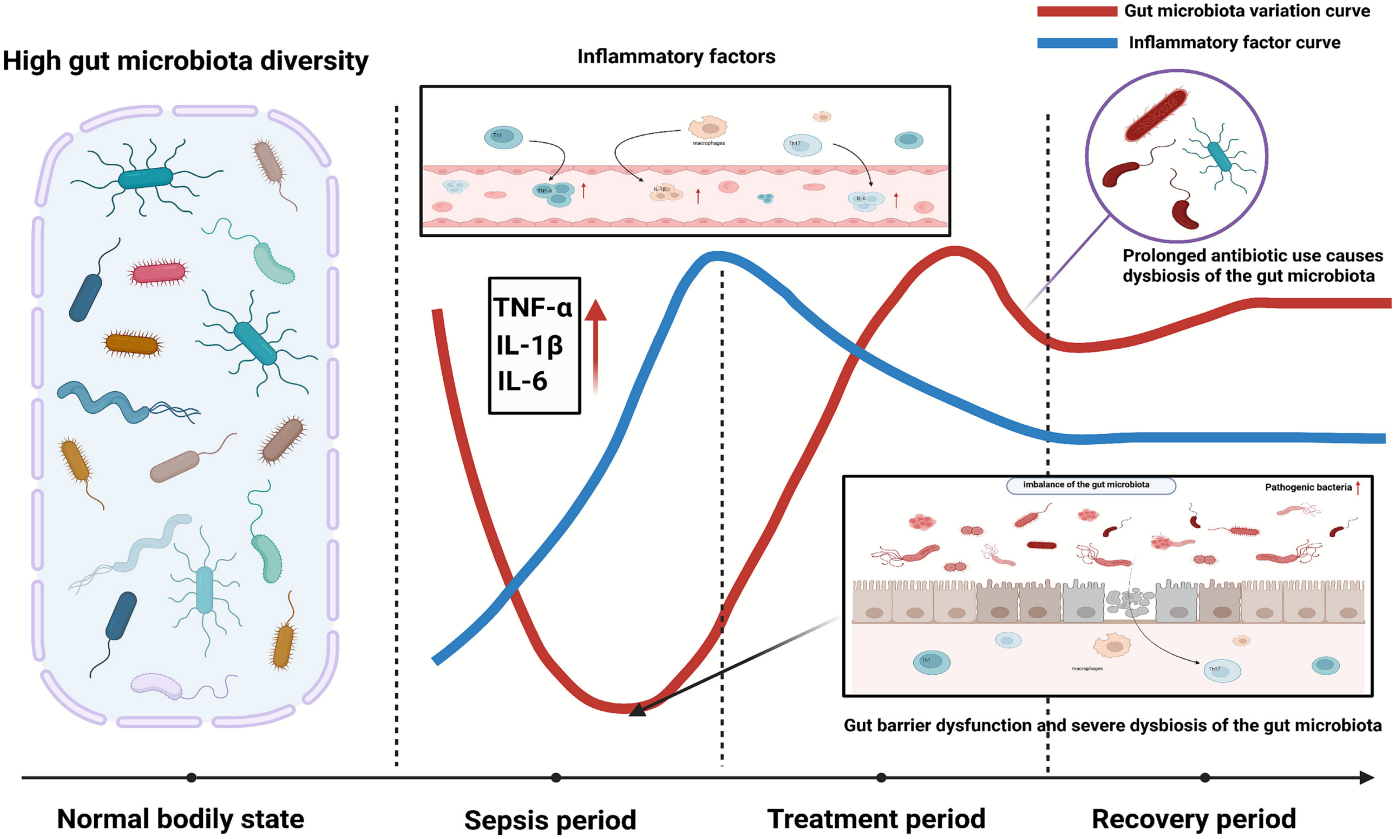
The changes in the gut microbiota before and after sepsis treatment.

How can we improve early diagnosis of GM dysbiosis in septic patients? How can we identify the optimal timing for intervention when dysbiosis begins to worsen? The treatment strategies outlined above may provide valuable insights for diagnosis and management. Most septic patients inevitably require antibiotics for infection control, so antibiotic usage and combinations should be at the core of diagnostic and therapeutic approaches. Firstly, regarding antibiotic administration, it is essential to precisely control dosage while ensuring an adequate treatment duration for sepsis patients. Regular sampling and analysis of the GM should be conducted, focusing on the abundance and changes in microbial composition, while minimizing interference with the GM. Adjustments to the types of antibiotics used should be made promptly to avoid the development of antibiotic-resistant bacteria. Additionally, probiotics or FMT could be introduced early after antibiotic administration, during the early stages of microbiota disruption, to enhance microbiota stability and slow or halt the progression of dysbiosis. It is crucial to emphasize that precise assessment of antibiotic dosage and type, combined with changes in the GM, is necessary for personalized intervention. This still requires validation through extensive animal studies and clinical trials. A single treatment approach may not be sufficient to meet the increasingly complex clinical needs of septic patients, and thus a multidimensional, integrated therapeutic strategy is recommended (Figure 3).

The gut microbiota exhibits a rich diversity under normal physiological conditions. However, as sepsis progresses, the gut microbiota gradually becomes imbalanced and disrupted, with inflammatory markers continuously rising. When the intestinal mucosal barrier is completely compromised, the gut microbiota reaches a nadir of imbalance. With the intervention of relevant therapies, the gut microbiota shows a trend of recovery, and inflammatory factors are gradually cleared. However, the long-term use of antibiotics still causes fluctuations in the microbial imbalance, until the immune function of the body is repaired, allowing the gut microbiota to eventually reach a stable state again.

## 4 Summary and outlook

As research into the role of GM in the pathogenesis of sepsis deepens, increasing evidence suggests that the imbalance of GM is closely associated with the onset and progression of sepsis. A decrease in microbial diversity and the overgrowth of pathogenic bacteria, particularly Gram-negative bacteria and pathogenic fungi, can damage the intestinal barrier, allowing harmful substances to enter the bloodstream. This activates systemic immune responses, triggers inflammatory syndromes, and exacerbates sepsis and organ dysfunction.

However, current research on the specific mechanisms of the GM in sepsis still faces many limitations. Firstly, although the potential mechanisms between the microbiome and sepsis have become a research hotspot, the specific roles of different microbiomes in sepsis remain unclear, and there is a lack of in-depth theoretical discussion. Current research mostly focuses on the impact of bacteria on the GM, overlooking the potential roles of viruses, *archaea*, bacteriophages, and other microorganisms in sepsis. This, to some extent, limits a comprehensive understanding of the mechanisms underlying GM imbalance. Secondly, existing clinical studies lack multi-center and large sample validation data, with most remaining at the animal model stage. This makes our understanding of the relationship between the GM and sepsis in patient populations somewhat one-sided and biased. Therefore, further research should focus on the roles of various microorganisms, conducting broader clinical trials and multi-center studies to better reveal the role and mechanisms of the GM in sepsis.

Current microbiota modulation strategies (e.g., probiotics, SCFAs) have only demonstrated theoretical potential in addressing sepsis-related gut dysbiosis, lacking sepsis-specific high-quality clinical evidence to support their effectiveness. Critically, probiotics suffer from the absence of standardized guidelines, leading to significant variations in efficacy due to inconsistent strains and dosages. Moreover, their regulatory effects are often short-lived and superficial, unable to restore the core diversity and functional integrity of the GM. The beneficial effects of SCFAs are largely confined to animal models, with a lack of clinical data and species-specific immune and microbiota differences hindering their clinical translation, exposing a significant gap between basic research and clinical needs. Overall, research on sepsis-related GM remains in its early exploratory stages, and has yet to bridge the critical gap from “correlation description” to “precise intervention.” The most promising therapeutic approach lies in personalized, combined microbiota modulation strategies: selecting probiotics based on patient microbiota profiling, optimizing short-chain fatty acid supplementation through targeted delivery technologies, or using FMT to achieve fundamental restoration of microbial homeostasis. This approach effectively circumvents the adaptability flaws and efficacy limitations of single-strategy interventions.

Key issues that must be prioritized in future research include: identifying the core functional targets and biomarkers of sepsis-specific GM dysbiosis, providing a precise basis for targeted therapies; establishing a standardized system for probiotics specifically tailored for sepsis, addressing clinical controversies related to strain selection, optimal dosage, and treatment duration; developing efficient targeted delivery systems for SCFAs to overcome the low bioavailability barrier; conducting large-scale multicenter RCTs to validate the efficacy and long-term safety of personalized modulation strategies, while clarifying their interactions and potential risks with conventional sepsis treatments; and deeply elucidating the core mechanisms through which microbiota modulation improves sepsis prognosis, moving beyond the “knowing what but not why” dilemma in current research.
